# Editorial: The Physiological Functions of the APP Gene Family

**DOI:** 10.3389/fnmol.2017.00334

**Published:** 2017-10-23

**Authors:** Ulrike C. Müller, Thomas Deller

**Affiliations:** ^1^Department of Functional Genomics, Institute for Pharmacy and Molecular Biotechnology, Universität Heidelberg, Heidelberg, Germany; ^2^Institute of Clinical Neuroanatomy, Neuroscience Center, Goethe University Frankfurt, Frankfurt, Germany

**Keywords:** amyloid precursor protein (APP), APP like proteins, synaptic plasticity, development, spine density, neuroprotection and neuronal repair, synaptogenesis, secretases

The amyloid precursor protein APP plays a key role in the pathogenesis of Alzheimer's disease (AD), as proteolytical cleavage of APP gives rise to the β-amyloid peptide Aβ, which is deposited in the brains of AD patients (Selkoe and Hardy, 2016). In contrast to this key role in AD, the reviews and original papers in this Special Issue entitled “The physiological functions of the APP gene family” aim to shed some light on the “bright side” of APP, which exhibits important physiological functions during brain development, for adult brain plasticity and protection against injury. This change of perspective is timely, since accumulating evidence suggests that disease symptoms are caused both by an overload of toxic substances, e.g., “too much Aβ,” as well as deficits of protective molecules, e.g., “not enough APPsα.”

Unraveling APP functions has not been trivial, since APP undergoes complex processing. APP processing is initiated either by α-secretase cleavage within the Aβ region, or by β-secretase (BACE) cleavage at the N-terminus of Aβ, leading to the secretion of large soluble ectodomains, termed APPsα and APPsβ, respectively. Subsequent processing of the C-terminal fragments (CTFα or CTFβ) by γ-secretase results in the production of Aβ, p3 and the APP intracellular domain (AICD). This processing—as well as processing along non-canonical pathways (see Müller et al., 2017, for review) results in numerous fragments, which have different and partially opposite functional properties. Furthermore, APP functions are in part shared by APP-like proteins 1 and 2 (APLP1 and 2), which confounds some experimental approaches. Finally, expression changes over time and with aging add additional levels of complexity. In short, understanding APP gene family functions is challenging and this special issue provides a broad overview of the state-of-the art in this field.

Several reviews (Seipold and Saftig; Endres and Deller; Yan; Becker-Pauly and Pietrzik) focus on the properties of canonical and non-canonical α-, and β-secretases, their substrates, regulation, and neurobiological functions in health and disease. Müller et al. give a systematic overview over proteomic methods to systematically identify the substrates of membrane proteases. The knowledge of these substrates is crucial to identify the physiological and pathological functions of secretases and to assess potential risks of their pharmacological impairment to treat AD (Endres and Deller; Yan). In addition, there is evidence that the secretases which are transmembrane proteases can form larger complexes with other cell surface proteins that may modulate their activity including members of the tetraspannin family (Seipold and Saftig). APP processing is further modulated by the lipid composition of the plasma membrane and accumulating evidence suggests that Aβ and the AICD play an important role in regulating lipid homeostasis (Grimm et al.). Likewise, lipoprotein receptors may interact with APP to control developmental processes and synaptic function (Pohlkamp et al.). They have been shown to not only regulate Aβ uptake and degradation, but also APP processing and APP trafficking. In this regard, employing live cell imaging in primary neurons Herr et al. demonstrate that low-density lipoprotein receptor-related protein 1 (LRP1) modulates the axonal transport of APP monomers and dimers.

There is a large body of evidence indicating that APP family proteins are multimodal proteins that can function as ligands via their secreted fragments, in particular APPsα, or as cell surface proteins mediating signal transduction and synaptic adhesion (as reviewed by Müller et al., 2017). Wild et al. discuss how metal (Cu and Zn) binding affects the structure of the APP extracellular domain and may modulate its role as a synaptic adhesion molecule. As APP family proteins have no enzymatic activities, signal transduction relies on interactions with other membrane proteins and/or adaptors. The role of the Fe65 adaptor family is summarized by Guenette et al. Fe65 binding to the APP C-terminus involves its phosphotyrosine-binding (PTB) domain 2 which can also mediate the formation of cytosolic Fe65 dimers, as shown by X-ray crystallography (Feilen et al.). The importance of heteromeric G-protein interactions with the APP C-terminus for physiological APP signaling and AD pathogenesis is reviewed by Copenhaver and Kogel.

Major insight into the *in vivo* functions of APP family proteins has been obtained from animal models. *Drosophila* expresses only one APP protein called APP-like (APPL) and two reviews (Cassar and Kretzschmar; Preat and Goguel) deal with APPL functions in flies. In mice the analysis of APP functions is complicated by partially overlapping functions within the gene family and lethality of double and triple knockout mice (Han et al.). To circumvent early postnatal lethality mice with conditional floxed alleles have been generated (Müller et al., 2017). Together, the analysis of engineered mouse models indicated that APP family proteins and their proteolytic fragments are important during nervous system development for neuronal migration, neurite outgrowth, axonal pathfinding, and synaptogenesis (Müller et al., 2017). Proteomic studies, reviewed by Weingarten et al. established APP family proteins as important components of the active zone. Lazarevic et al. demonstrated that low amounts of Aβ are involved in the regulation of neurotransmitter release. It should be noted, however, that APP family proteins have also been localized at postsynaptic sites including the neuromuscular junction. In addition, APP family proteins have important functions in the adult hippocampus, where they are differentially expressed in all subregions (Del Turco et al.) and regulate synaptic plasticity and memory (Ludewig and Korte). The recently identified function of APP for structural spine plasticity is summarized by Montagna et al. In particular APPsα holds great therapeutic potential for AD as reviewed by Mockett et al. Finally, Hefter and Draguhn highlight the role of APP and APPsα as a protective factor for acute neuronal insults including hypoxia.

We thank all contributors for their interesting and informative articles and the reviewers for their constructive and thoughtful suggestions.

## Author contributions

UM and TD wrote the manuscript and both authors approved the final version for publication.

### Conflict of interest statement

The authors declare that the research was conducted in the absence of any commercial or financial relationships that could be construed as a potential conflict of interest.
